# Decolonisation and Self-Regulation as Alternative Paths to Data Science Health Research Governance in Africa

**DOI:** 10.12688/wellcomeopenres.24070.1

**Published:** 2025-07-30

**Authors:** Oluchi C. Maduka, Simisola O. Akintola

**Affiliations:** 1 Department of Private and Property Law, University of Ibadan Faculty of Law, Ibadan, Oyo, 200005, Nigeria

**Keywords:** data science, health research, decolonisation, self-regulation, Africa, communitarianism, ethical governance, indigenous knowledge.

## Abstract

**Introduction:**

Data science health research (DSHR) presents new ethical challenges to the traditional model of human subject research, particularly by enabling data processing without the consent of data subjects. Although the current research governance framework makes informed consent a cornerstone of ethical research practices, obtaining individual consent can often be impractical in DSHR. This paper explores the alignment of DSHR with African customary governance and communal lifestyles as a framework for ethical research oversight.

**Methodology:**

Using a mixed-method approach, this study integrates doctrinal analysis of legal and policy frameworks with case studies from Nigeria, Kenya, Ghana, Uganda, and South Africa. Data were synthesised from peer-reviewed literature, with a focus on initiatives that operationalise decolonised governance.

**Results:**

Data science health research challenges traditional biomedical ethics by enabling data processing without consent, thereby questioning the longstanding principle that informed consent is a prerequisite for ethical research. However, this principle has been widely contested as a universal standard, particularly in African contexts where decision-making is often communal rather than individualistic. Case studies from Nigeria, Kenya, Ghana, Uganda and South Africa illustrate that while informed consent remains a normative requirement, largely to satisfy the expectations of funding bodies, communal approval is paramount. Furthermore, religious and cultural traditions often accommodate forms of paternalistic consent, reinforcing collective decision-making structures.

**Conclusion:**

Given that African societies emphasise communal governance, the ethical challenges posed by DSHR, particularly regarding consent, may be less pronounced in Africa. However, decolonisation and self-regulation are not merely theoretical constructs, but a practical and necessary process that requires deliberate action. Unless African leaders take decisive steps to restructure governance, prioritise self-reliance, and invest in homegrown research and development, the discourse on decolonising DSHR in Africa will remain purely theoretical, lacking the practical implementation needed for real change.

## Plain Language Summary

Although informed consent is the criterion for ethical research conduct, data science health research (DSHR) presents novel ethical challenges by enabling data processing without the consent of data subjects. This study introduced the decolonisation and self-regulation framework, a practice that researchers argue is inherent in most African societies, involving a more communal approach to collective decision-making.

Drawing on select case studies from Africa, this study identifies practices such as community engagement, community advisory boards, respect for elders and community leaders as interconnected elements that play a crucial role in facilitating informed consent in research. These practices can be leveraged to strengthen the ethical governance of DSHR across the continent.

To fully harness the vast potential of DSHR, its successful implementation is essential. Decolonisation and the self-regulation framework require deliberate actions. Further research could explore more case studies in diverse African sociopolitical contexts to assess best practices for effective implementation.

## Introduction

The rapidly evolving landscape of data science health research (DSHR) challenges the traditional notion of health research governance, one that has been shaped largely by Western-centric ideals of individual autonomy and informed consent ^1– 3^. Historically, this governance framework has been rooted in the ethical principle that individuals must provide explicit consent before their data can be used in research ^4– 6^. However, the increasing use of AI, machine learning (ML) tools, high-performance computing, and vast, diverse datasets indicates that it may be possible for health researchers to conduct data-driven studies without direct interaction with participants, thereby challenging this traditional paradigm ^3, 7^.

Over the past decade, African scholars have critically examined the applicability of this individualistic model within the African context. They argue that it does not represent the communitarian ethos that underpins African cultures, norms, and ethical perspectives ^8– 11^. In many African societies, decision-making and well-being are viewed through a collective lens rather than an individualistic one, suggesting that an alternative governance model, one that embraces communal values, may be more appropriate for regulating health research. Despite these arguments, the dominance of Western funding bodies in global health research has entrenched the individual autonomy model as the foundation of health research ethics, leaving little room for alternative frameworks to gain traction ^12^.

However, with the increasing complexity of data science methodologies, which often challenge the feasibility of obtaining traditional forms of consent, the communitarian approach long advocated by African scholars presents a viable alternative. This perspective supports a model of health research governance that prioritises collective well-being, shared decision-making, and trust, rather than rigidly adhering to an autonomy-driven framework that may not align with local cultural and ethical realities. Thus, as health research continues to evolve in the era of big data and AI, decolonising health research governance has become increasingly relevant. It necessitates a shift away from a one-size-fits-all model toward one that incorporates diverse ethical perspectives, particularly those that reflect the realities and values of African societies.

### Overview of data science health research

**Figure 1.**
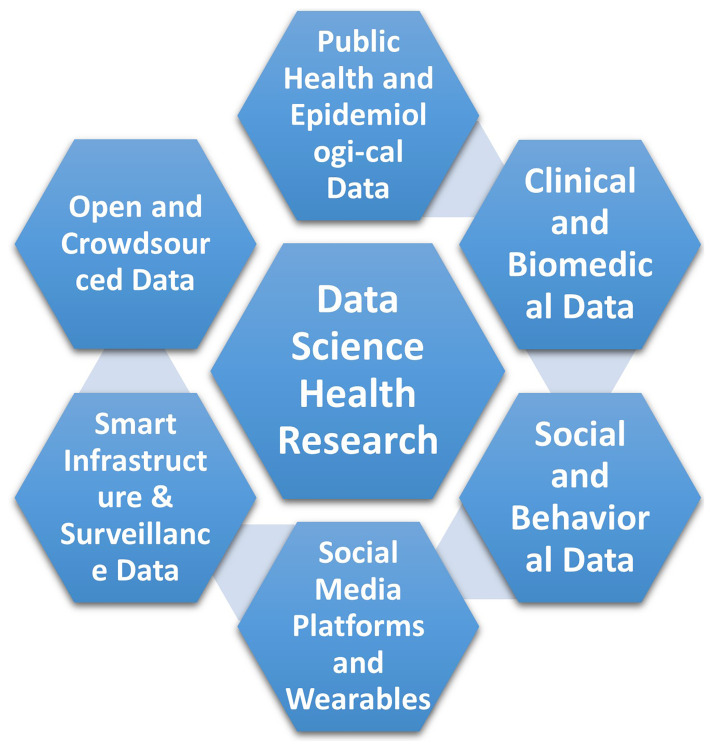
Different sources of data in data science health research.

According to the National Institutes of Health (NIH), data science is an “interdisciplinary field of inquiry in which quantitative and analytical approaches, processes, and systems are developed and used to extract knowledge and insights from increasingly large and/or complex sets of data” ^13^. Data science researchers utilise several computational techniques, tools, and methods such as Artificial Intelligence (AI), Machine Learning (ML), High-Performance Computing (HPC) and novel Algorithms to extract knowledge, meaningful insights, and innovations from complex and large datasets ^14– 16^. These datasets are diverse, ranging from conventional and unconventional sources, including public health and epidemiological data, clinical and biomedical data, as well as social media platforms and wearable devices. Figure 1 illustrates these diverse data sources.

In the health sector, for example, data scientists combine these tools and techniques to enhance data mining from health data and other diverse sources to enable improved understanding and management of diseases ^17– 21^. It is expected that the application of data science in health research will accelerate medical diagnosis ^22^, and improve personalized medicine ^23– 27^, and its ability to identify disease patterns could accelerate progress towards reducing disease burdens in Sub-Saharan Africa (SSA) in line with the 2030 Sustainable Development Goals ^28^. Presently, Africa bears 25% of the global disease burden ^29– 31^. With the right expertise and strategic application of data science in health research, Africa can develop tailored solutions to its unique challenges and pave the way for sustainable health development. However, the rapid growth of this field brings with it unique challenges.

The importance of health research governance cannot be overstated, and for data science health research, the existing governance may challenge the effective governance of DSHR in Africa. For instance, the existing health research governance structure makes informed consent mandatory ^32^. Justifiably as this may be, DSHR challenges the core notion of human participants in research by enabling data processing without the need for physical interaction with participants ^3^. The involvement of individuals in research without their knowledge undermines the core principle of informed consent. Additionally, the scale and intricacy of datasets often render the process of securing informed consent cumbersome or unfeasible ^33– 36^.

Within the past decades, some African scholars have begun to challenge the doctrine of informed consent as presently applied, arguing that African culture makes it impracticable. For example, Jegede discusses the inappropriateness of applying what he referred to as “Western bioethics” in African settings. According to Jegede, African ethics are deeply rooted in communalism, in contrast with Western individualism ^37^. As a result, adopting the Western concept of autonomy to research involving human subjects in Africa without understanding the community’s important role is improper. Jegede further argues that disregarding community engagement in health research in Africa can lead to ineffective management and a lack of properly informed consent ^37^. Furthermore, Retha Visagie *et al* argue that obtaining free, prior, and informed consent is deeply embedded in cultural norms. They contend that a uniform approach to informed consent disregards the perspectives of those involved ^38^. Instead, they advocate for an integrated strategy grounded in Afro-communitarianism, emphasising that social science researchers working with rural African communities must account for the values, concepts, and theories central to collective autonomy ^38^. In addition, Behrens challenges the Western notion of individual autonomy, highlighting that in many African contexts, community is highly valued, and individuals are profoundly ingrained in their communities. Behrens believes that the idea of ‘respect for autonomy’ is problematic in Africa due to its failure to acknowledge the intrinsic relationality of individuals ^39^. Several other authors share the same sentiments ^40– 42^.

In sum, Africa’s communitarian societal structure contrasts sharply with the West’s individualistic approach, leading many African scholars to argue that the current framework of informed consent may be impractical as a prerequisite for research on the continent. Various alternatives, such as multi-step consent, have been proposed to address this challenge. However, since health research funding predominantly originates from the Global North, informed consent continues to be enforced as a non-negotiable condition.

### Decolonisation

Decolonisation has been applied in various contexts, including health, education, politics, crime and justice, as well as governance, etc ^43^. The most common application pertains to the process by which colonies become independent of the colonising country ^44^. According to Ayana *et al*, decolonisation includes several political, social, economic and cultural aspects aimed at restoring autonomy, sovereignty and self-determination to formerly colonised territories and populations ^45^. Decolonisation was gradual and peaceful for some colonies largely settled by expatriates, but violent for others, where rebellions by the indigenes were energised by a sense of nationalism. Decolonisation requires dismantling deeply entrenched colonial systems, ideologies, narratives, identities, and practices while simultaneously fostering reconstruction aimed at reclaiming humanity, restoring physical integrity, and affirming self-determination ^45, 46^. Beyond achieving political and economic independence, which are key drivers of patriotic movements, it also involves addressing colonial legacies, including social injustices and cultural impositions ^47^.

Theories and frameworks such as the Fanonian approach, restorative justice, and the Monchalin approach have been used to understand and analyse the concept of decolonisation. According to Frantz Fanon, the proponent of the Fanonian approach, decolonisation not only means political freedom but also unlearning what had been taught, as well as building new identities ^48, 49^. Monchalin emphasises that decolonisation entails a profound transformation of colonial structures to address historical injustices and promote justice and peace through nation-to-nation partnerships ^50, 51^. Monchalin's perspective on decolonisation underscores the need for a fundamental shift in colonial structures and ideologies, particularly within justice systems, to address historical injustices and promote genuine reconciliation and peace ^52^. Indigenous decolonisation concepts focus on reclaiming self-determination, ancestral lands, and autonomous governance, drawing insights from the histories and resistance efforts of Indigenous communities worldwide. These perspectives challenge settler colonial rule, ecological destruction, and the loss of cultural identity, advocating for indigenous revival and the revitalisation of traditional knowledge systems ^53^. Restorative justice and decolonisation, on the other hand, are interconnected frameworks that aim to address historical injustices and promote healing and transformation within communities. The integration of decolonisation into restorative justice practices seeks to dismantle colonial structures and ideologies, particularly in justice systems that have historically marginalised Indigenous and other oppressed communities ^54, 55^.

### Decolonisation within the context of Global Health Research

The arguments on decolonisation have been applied within the context of global health research, especially since the COVID-19 pandemic ^56^. Issues about inequalities in resource allocation and expertise in data analysis, particularly in public health emergencies have raised issues of how colonisation has crippled Low and Middle-Income Countries (LMICs). These arguments suggest that it may be possible to have an African health research governance. Thus, any governance structure from the High-Income Countries (HIC) will be seen as a colonisation effort. Proponents of decolonising global health research argue for the dismantling of colonial influences in global health research. For example, Kumar *et al* argue that there exist power imbalances and resource disparities among global health actors, which often favour actors from the HIC over the LMIC ^57, 58^. Because of this advantage, actors from HICs dominate and shape global health structures, policies and practices, potentially sidelining the priorities and needs of the LMICs ^12^. Recently, some African researchers have argued that the exploitative practices of extracting resources and data from LMICs without adequate benefit to the local populations raise issues of data sovereignty ^59^. Consequently, decolonising global health will involve “removing all forms of supremacy within all spaces of global health practice within countries, between countries, and at the global level” ^60^.

### Self-Regulation

Throughout history, the everyday lives of most Africans have been governed by customary law, which is a system of rules recognised as binding by members of a community ^61, 62^. Although unwritten, customary law played a crucial role in maintaining social cohesion and ensuring prosperity ^63, 64^. Disputes within the community were resolved through witness testimony establishing the existence of customary practices. In rare cases, customary law judges acknowledged certain customs when they had become widely recognised and notorious ^65, 66^. By nature, African communities were self-regulatory and self-sufficient, which underscored the importance of kings, elders, and community leaders in decision-making ^67– 69^. This emphasis on communal governance also reinforced the prevalence of a communitarian lifestyle as the norm.

In this paper, we explored an alternative path to DSHR governance in Africa, which we termed decolonisation and self-regulation. Decolonisation is used not in terms of the historical colonial past, but rather to the act of rethinking and reshaping DSHR governance in Africa based on its unique societal, cultural, and ethical perspectives. We ask whether it is possible to approach data science health research from Africa’s unique perspective. Rather than adopting a framework predominantly shaped by Western paradigms, the paper proposes exploring whether Africa’s values, traditions, and communal priorities can inform and shape the governance of data science research in a manner that is both relevant and respectful to local contexts. This approach invites a shift from merely adapting external models to actively crafting governance frameworks that emerge organically from African societies themselves. Self-regulation extends beyond the conventional idea of simply adhering to externally imposed rules. It suggests a more autonomous and self-determined approach, where African nations and communities take responsibility for the governance and ethical oversight of DSHR. This perspective argues that Africa’s distinct cultural, ethical, and societal frameworks can provide an inherent capacity for self-governance that is both sustainable and culturally appropriate. Self-regulation implies that individuals can exercise a degree of control over their affairs, rather than relying solely on established legal rules. We argue that, even in the presence of legal frameworks, health researchers must demonstrate a level of control and self-restraint considering the complexities and ethical challenges in DSHR. This includes making informed decisions about the volume of data to use, determining when to anonymise data, and assessing whether publishing identifiable information is justified, particularly when it does not serve the public interest. This paper argues that such self-regulation is inherent in many African societies, which already have existing structures that influence and reinforce it.

### Materials and methods

This case study examines the research practices and frameworks of five African countries, each representing their respective regions and actively engaged in global research collaborations. Countries from North Africa were excluded due to language limitations, as some key materials were not available in English. The study draws on a diverse range of online resources and databases, including PubMed, Google Scholar, HeinOnline, and government websites. Additionally, it incorporates reviews of case law, social media pages, and external sources such as funders, networks, and scholarly articles.

### Current landscape of data science health research governance in Africa

The current landscape of data science health research governance in Africa is shaped largely by the already existing health research governance structures established over the past decades. Ethical frameworks, including Research Ethics Committees (RECs), have been instituted by many countries to review and oversee health research. RECs align their ethical review processes with international standards like the Declaration of Helsinki, the Council for International Organisations of Medical Sciences (CIOMS), the Belmont Report, the National Institutes of Health (NIH) Guidelines for the Conduct of Research Involving Human Subjects, as well as the World Health Organization (WHO) Operational Guidelines for Ethics Committees.

Research ethics regulations are fundamentally rooted in the Belmont Report, CIOMS Guidelines, and the Helsinki Declaration. These codes are anchored in the principle of autonomy, emphasising that individuals capable of making decisions must be allowed to do so, particularly in clinical care and research ^70^. Considering that the principle is usually a condition for the award of grants, this principle has been entrenched within the African research community. However, while the Belmont Report, CIOMS, etc. form the foundation of research frameworks in Africa, the emphasis on autonomy often challenges Africa's communal culture. Nevertheless, little is known about how this process is practically implemented as autonomy operates within a communal framework in most African communities. These practices, despite being criticised for limiting individual freedoms, are deeply embedded in cultural norms and values ^71– 73^. This cultural context contrasts starkly with practices in places like the United States, where autonomy is interpreted more individualistically. For instance, a decision that might seem routine in Northwestern Nigeria, such as deferring to the male head of the family, a husband or getting community approval, might be seen as outrageous in the more advanced economies such as the United States or the United Kingdom. This highlights the need to reconcile global research ethics principles with local cultural realities.

To address this gap, African researchers began to emphasise community engagement. According to Tindana *et al*, community engagement is the establishment of genuine partnerships characterised by mutual respect, inclusive participation, power sharing, equity, and shared benefits to seek a "win-win" outcome in collaborative initiatives ^74^. Community engagement is significant because it takes cognisance of community values, beliefs, and norms as they shape perceptions of study risks and benefits, influencing both independent decision-making and consent processes. Active community participation helps tailor consent procedures to local contexts, while engagement activities enhance communication, fostering respect, understanding of research implications, and acknowledgement of participants' contributions ^75, 76^.

Community engagement activities may also facilitate interpersonal communication, which is key to showing respect and making people understand the risks and benefits of the research, as well as appreciating participants' contributions ^77^. Through this approach, researchers argue that communities and their leaders must be involved and respected before any research is conducted, particularly in the demographic areas where the study will take place. Moreover, in an era of increasingly complex data ecosystems, where obtaining meaningful consent for online transactions is becoming impractical, the foundational emphasis on autonomy in research ethics guidelines warrants reconsideration. This is not a critique of autonomy but a call for reflection: if autonomy is the cornerstone of research ethics, it should not hinder the growth of DSHR. Researchers ought to insist on it even where the volume and complexities of the datasets present a challenge. However, the fact that one can argue that it can be dispensed with means that fundamentally, it is not an escape route ^7^. Africa’s experience suggests that the challenges autonomy presents in some communities should inform more inclusive frameworks.

Secondly, assuming that research ethics guidelines were fundamentally developed in Africa, would the concept of informed consent have evolved differently? While respecting research participants would remain a priority, the process might reflect communal values rather than the purely individualistic approach of the current model. Research suggests that acquiring voluntary consent in African cultures can be difficult, requiring more than just providing information and securing consent ^78– 82^. Religion, language hurdles, literacy levels, power disparities, and socio-cultural norms and beliefs can limit individuals' ability to freely agree to participate in research ^83– 85^. These issues are exacerbated where research is conducted in communities with strong ties to religious and cultural norms or where teenagers are actively involved in the research ^86– 88^.

Finally, while scholars worldwide grapple with the challenges posed by DSHR, there is much to learn from Africa. Despite decades of adhering to external research ethics frameworks, African scholars have worked to preserve their cultural norms and practices through self-governance. This unique approach could serve as a model for integrating diverse perspectives into global research ethics frameworks.

### Case studies of decolonised research models in Africa

Within Africa, over 40 countries have adopted some form of research ethics codes, largely reflecting global standards such as the Belmont Report, CIOMS Guidelines, and the Helsinki Declaration. However, a select few countries and initiatives have begun incorporating localised and decolonised approaches into their health research frameworks. This section highlights and discusses examples of these efforts.

***The San People of Southern Africa.*** The San people of Southern Africa, also known as Bushmen, are one of the world’s oldest continuous cultures, with their history stretching back tens of thousands of years ^89^. They are indigenous to Southern Africa, with a cultural identity identified as hunter-gatherers, a shared ancestry that has now been confirmed by genetic research ^90^. Because they are the oldest genetic ancestors of modern humans, they have for years been the focus of health research ^91^. San leaders have often felt dissatisfied and distasteful about some of the research and conclusions reached, especially when the San people were the subjects of research ^92^. Consequently, in 2017, through strong leadership, the San leaders of Southern Africa developed the San Code of Research Ethics, the first of such in Africa. The key point contained in this Code is that researchers engaging with San communities must uphold the principles of fairness, respect, care, and honesty while also obtaining community approval through a simple process of community structures that were put in place ^93, 94^. The San Code emphasises respect not only for individuals but also for the community, culture, and history. It specifically highlights past instances where San leaders were disregarded and excluded from the research process. The Code calls for open and transparent communication between researchers and San leaders, ensuring meaningful engagement. More so, it stresses that research should align with local needs and contribute to improving the lives of the San people. This care must extend to the families of those involved, as ethical research should recognise and respect the San people's identity while adhering to the cultural and social principles outlined in the Code of Ethics.

***Kenya.*** Kenya has implemented frameworks like the Kenya Medical Research Institute Guidelines (KEMRI), which prioritise local involvement and community consultation in health research.

Ethical approvals in Kenya often require researchers to demonstrate how they have engaged with local communities, reflecting an effort to align research with the values and needs of Kenyan society. For example, Section 9.1 of the KEMRI mandates investigators to ensure that community engagement, recruitment methods and advertisement for enrolment in a study are done in an ethically acceptable manner and that materials and methods used in recruitment are approved by KEMRI SERU ^95^. The guidelines equally made provisions for the community advisory boards, a group that presents the interests of the community concerning the planned research to the Principal Investigator (PI). The PI is expected to define their community engagement plan in the study protocol and recruitment procedures. This will document relevant stakeholders, how potential participants will be approached, incentives to be given, and materials and advertisements to be used. Most importantly, Section 11.1 of the guidelines provides that to mitigate the risk of the research, community leaders must be involved throughout the regular meetings of the research.

In other words, traditional structures, such as elders and community councils, are involved in the research process. For research conducted among indigenous communities or in rural areas, researchers are required to obtain the communal consent of leaders, elders, or councils before engaging individuals. This practice suggests that obtaining communal consent from leaders, elders, or councils is crucial for ethical research with indigenous communities where community decision-making is prioritised over individual autonomy. This also emphasises the importance of cultural sensitivity and collaboration within these communities ^96– 98^.

***Nigeria.*** Nigeria, the most populous country in Africa, is a nation of immense cultural and religious diversity. With over 250 native languages and multiple religious affiliations, health research in the country has often been fraught with controversy. However, since the introduction of the Health Research Ethics Code in 2007, greater coordination and orderliness have been brought to health research governance. As a predominantly paternalistic society, with over 50% of the population identifying as Muslim, traditional consent requirements have historically posed challenges ^99^. Individual consent for women was particularly difficult to implement, as male family members customarily provided consent on behalf of their wives, and fathers did so for their unmarried daughters ^100, 101^. This practice raises important considerations in the context of global health research ethics. The principle of autonomy asserts that individuals with decision-making capacity should be allowed to make choices concerning themselves. While religious and cultural factors may influence consent practices, a critical question arises: if a woman willingly permits her husband to make decisions on her behalf, has the woman exercised her autonomy? In a broader sense, if a community collectively agrees that a "king" should make decisions on their behalf based on their trust and belief in the ruler's character, can this be considered an exercise of their autonomy? These distinct African perspectives, although diverging from Western ideals, warrant further engagement and recognition within global health research.

More broadly, the Nigerian Code of Health Research Ethics has institutionalised community engagement, mandating that researchers initiate discussions with communities before conducting studies. Ironically, this requirement places significant decision-making power in the hands of community leaders, who determine which research projects are acceptable for their members. To facilitate dialogue between community members, research participants, and researchers, the Code of Health Research Ethics provides for the establishment of Community Advisory Committees (CACs). These committees play a vital role in ensuring that community concerns are integrated into research activities. Some of their functions include relaying community concerns and issues to leaders, research teams, institutional officials, or the HREC, advising on key aspects of the informed consent process and assisting with participant recruitment and retention ^102^. By incorporating these mechanisms, the Research Ethics Code seeks to balance respect for cultural norms with ethical research practices, ensuring that health research in Nigeria remains locally relevant.

***Ghana.*** Ghana has actively implemented communal consent and community engagement strategies in health research, aligning research practices with traditional authority structures to ensure cultural appropriateness and community involvement. In northern Ghana, the Navrongo Health Research Centre (NHRC) has effectively integrated traditional community practices with modern research methodologies. By engaging local chiefs and elders through customary gatherings such as “durbars” the NHRC facilitates public discussions about proposed research projects. This approach not only respects traditional leadership but also fosters community trust and minimises potential ethical issues, thereby enhancing the ethical conduct of research. Similarly, the Kintampo Health Research Centre (KHRC) in central Ghana has prioritised community engagement as a core aspect of its research endeavours ^103^. The KHRC involves community members in defining research agendas and seeks their input throughout the research process. This participatory approach ensures that studies address local health priorities and that findings are more readily accepted and implemented by the community ^103^. Additionally, the Global Health Research Unit on Global Surgery (GSU) in Ghana has collaborated with local communities to inform surgical research. By engaging hernia patients, community leaders, and other stakeholders in rural areas, the GSU has gathered valuable insights into the relevance, acceptability, and feasibility of clinical trials. This engagement has led to protocol adjustments that better reflect community needs and preferences ^104^. These initiatives underscore Ghana's commitment to integrating communal consent and community engagement into health research, ensuring that studies are culturally sensitive, ethically sound, and aligned with the health needs of its diverse populations.

***Uganda.*** Studies have shown that since gaining independence in 1962, Uganda has made concerted efforts to decolonise not only its political structures but also its health system governance ^105^. Scholars widely agree that colonial legacies in Uganda negatively impacted traditional forms of community participation in health system governance ^106, 107^. However, recent event shows that there are efforts to go back to the precolonial era. For example, the government is actively supporting traditional medicine practitioners, recognising their role in the health system, a practice that was previously suppressed under colonial rule. A significant turning point was the landmark case of *Salvatori Abuki and Richard Abuga v. Attorney General ^108^*, where the court declared unconstitutional the laws that criminalised traditional medicine practitioners under the guise of the Witchcraft Suppression Act. Additionally, initiatives such as *Bulungi Bwansi,* a self-help communal work system for public welfare, fostering collective action, traditional community engagement, and broader community participation, have been reintegrated into health system governance ^106^. Section 3 of the National Guidelines for Research Involving Humans as Research Participants (the Guidelines) makes provision for a Community Advisory Board, which must include members such as religious leaders, community leaders, as well as individuals with an understanding of local laws, cultural laws and gender issues. The main function of the Community Advisory Board is to assist investigators in understanding and incorporating community concerns into their research activities. Although the Guidelines specifically provide that community involvement should not override voluntary informed consent, the community advisory boards are empowered to advise on issues central to informed consent ^109^.

## Discussion of case studies

Over the past three decades in Africa, research ethics regulations have proliferated, with a strong emphasis on respecting research participants. The majority of African countries have established ethics codes, largely adapted from international frameworks such as the Belmont Report, CIOMS and the Declaration of Helsinki. Informed consent is a key requirement, and ethics committees play a crucial role in ensuring its implementation. However, while informed consent is considered a fundamental prerequisite for ethical research, it remains unclear how this consent is obtained in practice, particularly in rural communities where a strong emphasis in community heads is key.

The San Code of Research Ethics from the Southern Africa region emphasise community approval and respect for community and culture. For the San people of Southern Africa, meaningful engagement entails open and transparent communication between researchers and San leaders. The Code highlights that, in the past, the San people were often confused and addressed in complex scientific language they could not easily understand. Consequently, they expect researchers to treat them with respect in communicating in the language they understand. This language barrier was equally exemplified in Nigeria where it was reported that one of the reasons Nigeria failed to meet its Millennium Development Goals was the lack of communication in indigenous languages. Key informational booklets were not translated, making it difficult for rural communities who needed them the most to understand what was written and what was expected of them ^110^. This further emphasises that, regardless of the good intentions behind global health practices, they must align with the cultural norms and values of the communities they aim to serve.

Nigeria's advisory committees, as well as the Kenya advisory boards, show an effort to align health research with the values and norms of the community by deferring to a group of experts who are closely connected to the people. Specifically, the KEMRI notes that to mitigate risk, community leaders must be involved throughout the regular meetings of the research. In northern Ghana, the practice of engaging local chiefs and elders through customary gatherings is well recognised. This aims to respect traditional leadership and foster community trust. Unlike in other places in Africa where researchers through academic writings are making cases for decolonising health research governance, Uganda is one place where the national government and other arms of government have taken active and concrete steps through case law, amendment of laws and initiatives such as the *Bulungi Bwansi*.

In addition to these specific case studies, some other African countries have also tried to address ethical issues in global health research within a local context. The South African Good Clinical Practice Guidelines emphasise community engagement, cultural sensitivity, and respect for local norms. The country's health research ethics framework, overseen by the National Health Research Ethics Council (NHREC), incorporates principles that encourage the inclusion of local communities in decision-making processes ^111^. Research involving indigenous knowledge, such as traditional medicine, is regulated to ensure the respect and protection of cultural heritage, moving toward decolonised practices. In Botswana, the involvement of tribal authorities and community representatives in research approval processes highlights a communal approach. Tribal leaders must provide consent on behalf of their communities before research can proceed ^112^. Individual consent is secondary to this broader community consent, especially in rural areas where decisions are deeply rooted in communal traditions. The Botswana Health Research and Development Committee requires researchers to ensure that health research reflects the needs of the local population. Botswana has also integrated traditional leadership structures into research approval processes, which helps balance global research standards with local cultural norms.

## Conclusion

The evolving landscape of DSHR calls for a governance model that is both ethically sound and culturally relevant. While the principle of informed consent has been upheld as a cornerstone of global health research ethics, its strong emphasis on individual autonomy reflects a distinctly Western philosophical tradition. In contrast, African societies, which have long embraced communitarian values, naturally may accommodate other consent models. The challenges posed by data science research, particularly regarding the feasibility of obtaining individual consent in large-scale data use, further expose the limitations of a one-size-fits-all ethical framework. However, these challenges should not be viewed as obstacles but rather as an opportunity for African nations to fashion out governance structures that align with their indigenous ethical perspective. The regulatory frameworks emerging in countries such as Nigeria, Ghana, Kenya, and Uganda, in addition to the San Code of Research Ethics in Southern Africa, demonstrate a shift toward decolonised and self-regulated approaches that prioritise community engagement, respect for elders, and the role of local gatekeepers.

Crucially, the dominance of individual consent in African health research governance has been largely sustained by the funding priorities of the Global North, rather than by an organic alignment with African ethical perspectives. Moving forward, African nations must continue to challenge and reshape research ethics frameworks in ways that uphold both technological advancements and indigenous governance models. By embracing self-regulation and community-centred ethical standards, Africa has the potential to lead a transformative shift in global research ethics, which acknowledges multiple pathways to ethical legitimacy rather than imposing singular, Western-based philosophical traditions. A decolonial and plural approach to informed consent and research ethics in global health can enhance social justice and accountability for all stakeholders ^113^.

## Limitations of this study

Decolonisation is not merely a theoretical concept, but a practical and necessary process that requires deliberate action. Those committed to decolonisation must take proactive steps to address the challenges that arise when dependence on Western structures is rejected. However, African governments have repeatedly demonstrated an inability or unwillingness to engage in meaningful discussions and initiatives on decolonisation. Despite the continent's vast resources, many political leaders continue to rely on foreign aid and grants from Western nations rather than pursuing innovative, homegrown solutions to economic challenges. Instead of fostering self-sufficiency, this dependency perpetuates poverty and hinders sustainable development. While private institutions and researchers are making commendable efforts to advance the decolonisation agenda, policy discussions in this area ultimately require political will. Since legal frameworks largely dictate national policies and institutional practices, any substantial progress must be driven by legislative and governmental action. Unless African leaders take decisive steps to restructure governance, prioritise self-reliance, and invest in homegrown research and development, the discourse on decolonising health research in Africa will remain purely theoretical, lacking the practical implementation needed for real change.

Secondly, this study acknowledges that limiting the analysis to five countries out of Africa’s 54 may not fully capture the continent's diverse health research governance landscapes.

## Further research

While this paper outlines the need for decolonised approaches to health research governance, particularly as it concerns DSHR, further research is required to develop concrete methodologies and procedures for implementing these proposals effectively. Further research could explore more case studies within diverse African sociopolitical contexts to assess best practices for integrating Africa’s unique communitarian ethos into existing regulatory structures in a way that is globally accepted and ethical.

## Ethical approval

This research study did not involve human participants, identifiable personal data, or biological samples and therefore did not require ethical approval.

## Consent

N/A

## Data Availability

The data for this article consists of bibliographic references, which are included in the References section. N/A

## Supplementary materials

N/A
