# Decolonisation and Self-Regulation as Alternative Paths to Data Science Health Research Governance in Africa

**DOI:** 10.12688/wellcomeopenres.24070.2

**Published:** 2025-12-11

**Authors:** Oluchi C. Maduka, Simisola O. Akintola

**Affiliations:** 1 Department of Private and Property Law, University of Ibadan Faculty of Law, Ibadan, Oyo, 200005, Nigeria

**Keywords:** data science, health research, decolonisation, self-regulation, Africa, communitarianism, ethical governance, indigenous knowledge.

## Abstract

**Introduction:**

Data science health research (DSHR) presents new ethical challenges to the traditional model of human subject research, particularly by enabling data processing without the consent of data subjects. Although the current research governance framework makes informed consent a cornerstone of ethical research practices, obtaining individual consent can often be impractical in DSHR. This paper explores the alignment of DSHR with African customary governance and communal lifestyles as a framework for ethical research oversight.

**Methodology:**

This paper adopts a case study methodology, using a comparative analysis of decolonisation and self-regulation in health research across five African countries—Nigeria, Kenya, Ghana, Uganda, and South Africa. The study combines doctrinal analysis of legal and policy frameworks with reviews of peer-reviewed literature, case law, and diverse online resources such as PubMed, Google Scholar, HeinOnline, and government websites.

**Results:**

Data science health research challenges traditional biomedical ethics by enabling data processing without consent, thereby questioning the longstanding principle that informed consent is a prerequisite for ethical research. However, this principle has been widely contested as a universal standard, particularly in African contexts where decision-making is often communal rather than individualistic. The case studies illustrate that while informed consent remains a normative requirement, largely to satisfy the expectations of funding bodies, communal approval is paramount. Furthermore, religious and cultural traditions often accommodate forms of paternalistic consent, reinforcing collective decision-making structures.

**Conclusion:**

Given that African societies emphasise communal governance, the ethical challenges posed by DSHR, particularly regarding consent, may be less pronounced in Africa. However, decolonisation and self-regulation are not merely theoretical constructs, but a practical and necessary process that requires deliberate action. Unless African leaders take decisive steps to restructure governance, prioritise self-reliance, and invest in homegrown research and development, the discourse on decolonising DSHR in Africa will remain purely theoretical, lacking the practical implementation needed for real change.

## Introduction

The rapidly evolving landscape of data science health research (DSHR) challenges traditional ethical frameworks of health research governance, which emphasises individual autonomy and informed consent as their core principles ^1– 3^. Historically, this framework has been rooted in the principle that individuals must provide explicit consent before their data can be used in research ^4– 6^. However, the increasing use of artificial intelligence (AI), machine learning (ML) tools, high-performance computing (HPC), and vast, diverse datasets indicates that it may be possible for health researchers to conduct data-driven studies without direct interaction with participants, thereby challenging this traditional paradigm ^3, 7^.

Over the past decade, some African scholars have critically examined the applicability of this individualistic model within the African context. They argue that it does not represent the communitarian ethos that underpins African cultures, norms, and ethical perspectives ^8– 11^. In many African societies, decision-making and well-being are viewed through a collective lens rather than an individualistic one, suggesting that an alternative governance model, one that embraces communal values, may be more appropriate for regulating health research. Despite these arguments, the dominance of Western funding bodies in global health research has entrenched the individual autonomy model as the foundation of health research ethics, leaving little room for alternative frameworks to gain traction ^12^.

However, with the increasing complexity of data science methodologies, which often challenge the feasibility of obtaining traditional forms of consent, the communitarian approach long advocated by African scholars presents a viable alternative. This perspective supports a model of health research governance that prioritises collective well-being, shared decision-making, and trust, rather than rigidly adhering to an autonomy-driven framework that may not align with local cultural and ethical realities. Thus, as health research continues to evolve in the era of big data and AI, decolonising health research governance has become increasingly relevant. It necessitates a shift away from a one-size-fits-all model toward one that incorporates diverse ethical perspectives, particularly those that reflect the realities and values of African societies.

### Overview of data science health research

**Figure 1.**
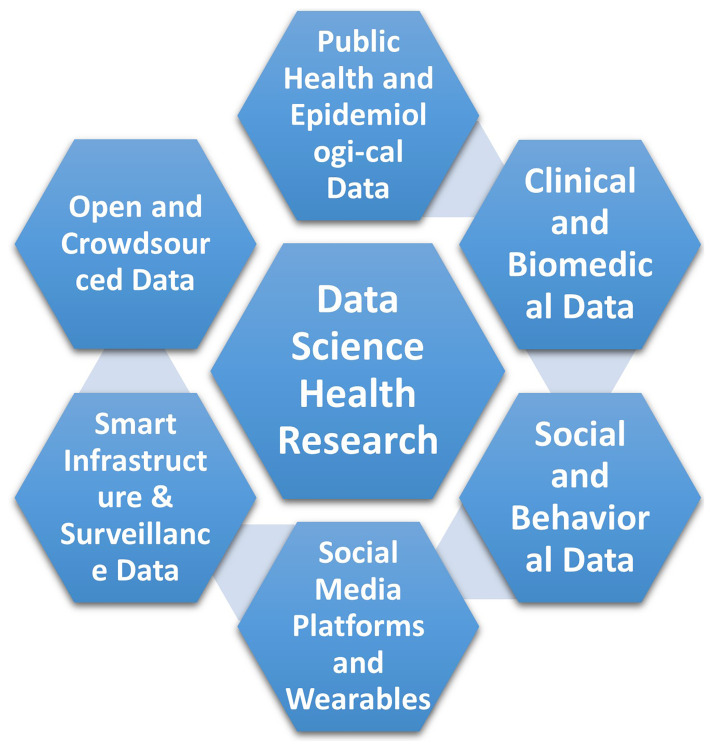
Different sources of data in data science health research.

According to the National Institutes of Health (NIH), data science is an “interdisciplinary field of inquiry in which quantitative and analytical approaches, processes, and systems are developed and used to extract knowledge and insights from increasingly large and/or complex sets of data” ^13^. Data science researchers utilise several computational techniques, tools, and methods such as AI, ML, HPC and novel Algorithms to extract knowledge, meaningful insights, and innovations from complex and large datasets ^14– 16^. These datasets are diverse, ranging from conventional and unconventional sources, including public health and epidemiological data, clinical and biomedical data, as well as social media platforms and wearable devices. Figure 1 illustrates these diverse data sources.

In the health sector, for example, data scientists combine these tools and techniques to enhance data mining from health data and other diverse sources to enable improved understanding and management of diseases ^17– 20^. It is expected that the application of data science in health research will accelerate medical diagnosis ^21^, and improve personalized medicine ^22– 26^, and its ability to identify disease patterns could accelerate progress towards reducing disease burdens in Sub-Saharan Africa in line with the 2030 Sustainable Development Goals ^27^. Presently, Africa bears 25% of the global disease burden ^28– 30^. With the right expertise and strategic application of data science in health research, Africa can develop tailored solutions to its unique challenges and pave the way for sustainable health development. However, the rapid growth of this field brings with it unique challenges.

Traditional biomedical research, such as clinical trials, typically involves recruiting participants through direct interaction and obtaining their informed consent ^31^. By contrast, DSHR depends on complex, large-scale, and diverse datasets, often without any direct engagement with individuals. It frequently entails the secondary use and reuse of data, as well as large-scale transfers across institutions and national borders, raising new challenges for data governance and ethical oversight ^14^. Advanced computational techniques, including ML, can link disparate data points in ways that may expose individuals or groups who would not otherwise consent to participation, raising concerns about collective harm and the stigmatisation of specific communities ^32, 33^. Moreover, the population-level effects of algorithmic outputs, particularly when they shape public health policy or clinical practice, highlight the limits of individual consent and the need for community-level governance ^32^. Unlike conventional research projects with clearly defined endpoints, DSHR systems operate through continuous learning, evolving as new data are incorporated. This perpetual development cycle calls for ongoing oversight and adaptive governance mechanisms rather than the one-time consent models that have historically structured human subjects’ research.

Over the past decade, other forms of research methods, such as genomics and stem cell research have challenged traditional informed consent models given their opaque nature, leading to the birth of other consent models such as the broad and tiered consent. More so, several regulatory provisions like the United States Code of Federal Regulations in 45 C.F.R. § 46.116(c) permit consent waivers under specific conditions. These waivers are justified in circumstance where obtaining consent may be impracticable, where the research poses minimal risks, and where participants rights and welfare are not likely to be jeopardised. Notwithstanding these waivers, informed consent remains the normative foundation of ethical research. In contrast, however, DSHR renders informed consent impossible *abi initio*. Researchers working with complex, large volumes and tens of varieties of datasets recognise that individual level consent is impracticable, cumbersome and impossible to secure ^34– 37^. Yet, informed consent remains deeply entrenched as the standard framework for ethical research. Justifiably as this may be, DSHR challenges this by enabling data processing without the need for physical interaction with participants. The possibility that individuals may be involved in research without their informed consent undermines the core principle of informed consent. In this regard, DSHR presents governance challenges that challenge existing frameworks, underscoring the need for alternative ethical approaches. Because of the scale and complexity of big data analytics, it is often unrealistic to obtain consent from every individual whose data is involved. In such circumstances, community-level consent may provide a more viable alternative. This challenge becomes more pronounced when considering that data science frequently involves repurposing existing data for new research questions, a practice that challenges traditional consent models while aligning more naturally with communal frameworks that prioritise broader community benefit over individual permission.

Within the past decades, some African scholars have begun to challenge the doctrine of informed consent as presently applied, arguing that African culture makes it impracticable. For example, Jegede discusses the inappropriateness of applying what he referred to as “Western bioethics” in African settings. According to Jegede, African ethics are deeply rooted in communalism, in contrast with Western individualism ^38^. As a result, adopting the Western concept of autonomy to research involving human subjects in Africa without understanding the community’s important role is improper. Jegede further argues that disregarding community engagement in health research in Africa can lead to ineffective management and a lack of properly informed consent ^38^. Furthermore, Retha Visagie *et al.* argue that obtaining free, prior, and informed consent is deeply embedded in cultural norms. They contend that a uniform approach to informed consent disregards the perspectives of those involved ^39^. Instead, they advocate for an integrated strategy grounded in Afro-communitarianism, emphasising that social science researchers working with rural African communities must account for the values, concepts, and theories central to collective autonomy ^39^. In addition, Behrens challenges the Western notion of individual autonomy, highlighting that in many African contexts, community is highly valued, and individuals are profoundly ingrained in their communities. Behrens believes that the idea of ‘respect for autonomy’ is problematic in Africa due to its failure to acknowledge the intrinsic relationality of individuals ^40^. Appaih *et al.* ^40^ finds that the individual-based informed consent model in Africa is incompatible with social values and undermines community gatekeepers' authority. Appiah *et al.* recommend, among other measures, the development of an African-centered model to address the limitations of how informed consent is currently applied on the continent. Several other authors share the same sentiments ^41– 43^.

 However, while African communitarian philosophies provide particularly rich frameworks for rethinking consent, communitarian and relational approaches are not unique to Africa. African indigenous governance traditions align with global data governance frameworks such as Collective Benefit, Authority to Control, Responsibility, Ethics, (CARE) particularly in asserting cultural sovereignty and emphasising relational ontologies that understand individuals as embedded within community relationships ^44^. Comparable commitments are evident in Indigenous models worldwide, for example, among Aboriginal Australians, Māori in New Zealand, and First Nations in Canada, which operationalise cultural sovereignty and relational ethics through collective decision-making and data governance ^45, 46^. Even within Western contexts, initiatives such as Patient and Public Involvement (PPI) in the United Kingdom and Community and Consumer Involvement (CCI) in Australia represent attempts to reconcile individual autonomy with communal participation in research governance ^47^.

Yet, African philosophies, however, contribute unique frameworks that move beyond existing models. For instance, the South African *Ubuntu* philosophy, with its foundational principle that “ *I am because we are*,” frames interdependence not as a social preference but as an ontological reality underpinning moral reasoning ^48– 50^. Likewise, the Yoruba concept of Omoluabi in Nigeria, literally translated as ‘child of noble character’ provides a complementary framework emphasising moral character, integrity, and accountability to community welfare. In research governance contexts, Omoluabi principles require that researchers and community leaders demonstrate not only procedural compliance but also moral excellence in ensuring that decisions genuinely serve communal interests ^51, 52^. Taken together, Ubuntu establishes the relational foundation for collective decision-making while Omoluabi provides ethical standards by which decisions are evaluated. These African philosophical traditions are embedded within indigenous political structures such as councils of elders and community assemblies, creating governance models that are not merely parallel to Western ethics but deeply integrated into existing cultural systems. This dual emphasis on relationality and character-based leadership differentiates African frameworks from other communitarian models that may prioritise collective benefit while maintaining individualistic assumptions about human nature or procedural safeguards alone.

### Decolonisation

Decolonisation has been applied in various contexts, including health, education, politics, crime and justice, as well as governance, etc ^53^. The most common application pertains to the process by which colonies become independent of the colonising country ^54^. According to Ayana *et al.*, decolonisation includes several political, social, economic and cultural aspects aimed at restoring autonomy, sovereignty and self-determination to formerly colonised territories and populations ^55^. Decolonisation was gradual and peaceful for some colonies largely settled by expatriates, but violent for others, where rebellions by the indigenes were energised by a sense of nationalism. Decolonisation requires dismantling deeply entrenched colonial systems, ideologies, narratives, identities, and practices while simultaneously fostering reconstruction aimed at reclaiming humanity, restoring physical integrity, and affirming self-determination ^55, 56^. Beyond achieving political and economic independence, which are key drivers of patriotic movements, it also involves addressing colonial legacies, including social injustices and cultural impositions ^57^.

Theories and frameworks such as the Fanonian approach, restorative justice, and the Monchalin approach have been used to understand and analyse the concept of decolonisation. According to Frantz Fanon, the proponent of the Fanonian approach, decolonisation not only means political freedom but also unlearning what had been taught, as well as building new identities ^58, 59^. Monchalin emphasises that decolonisation entails a profound transformation of colonial structures to address historical injustices and promote justice and peace through nation-to-nation partnerships ^60, 61^. Monchalin's perspective on decolonisation underscores the need for a fundamental shift in colonial structures and ideologies, particularly within justice systems, to address historical injustices and promote genuine reconciliation and peace ^62^. Indigenous decolonisation concepts focus on reclaiming self-determination, ancestral lands, and autonomous governance, drawing insights from the histories and resistance efforts of Indigenous communities worldwide. These perspectives challenge settler colonial rule, ecological destruction, and the loss of cultural identity, advocating for indigenous revival and the revitalisation of traditional knowledge systems ^63^. Restorative justice and decolonisation, on the other hand, are interconnected frameworks that aim to address historical injustices and promote healing and transformation within communities. The integration of decolonisation into restorative justice practices seeks to dismantle colonial structures and ideologies, particularly in justice systems that have historically marginalised Indigenous and other oppressed communities ^64, 65^.

### Decolonisation within the context of Global Health Research

The arguments on decolonisation have been applied within the context of global health research, especially since the COVID-19 pandemic ^66^. Issues about inequalities in resource allocation and expertise in data analysis, particularly in public health emergencies have raised issues of how colonisation has crippled Low and Middle-Income Countries (LMICs). These arguments suggest that it may be possible to have an African health research governance. Thus, any governance structure from the High-Income Countries (HIC) will be seen as a colonisation effort. Proponents of decolonising global health research argue for the dismantling of colonial influences in global health research. For example, Kumar *et al.* argue that there exist power imbalances and resource disparities among global health actors, which often favour actors from the HIC over the LMIC ^67, 68^. Because of this advantage, actors from HICs dominate and shape global health structures, policies and practices, potentially sidelining the priorities and needs of the LMICs ^12^. Recently, some African researchers have argued that the exploitative practices of extracting resources and data from LMICs without adequate benefit to the local populations raise issues of data sovereignty ^69^. Consequently, decolonising global health will involve “removing all forms of supremacy within all spaces of global health practice within countries, between countries, and at the global level” ^70^.

### Self-Regulation

Throughout history, the everyday lives of most Africans have been governed by customary law, which is a system of rules recognised as binding by members of a community ^71, 72^. Although unwritten, customary law played a crucial role in maintaining social cohesion and ensuring prosperity ^73, 74^. Disputes within the community were resolved through witness testimony establishing the existence of customary practices. In rare cases, customary law judges acknowledged certain customs when they had become widely recognised and notorious ^75, 76^. By nature, African communities were self-regulatory and self-sufficient, which underscored the importance of kings, elders, and community leaders in decision-making ^77– 79^. This emphasis on communal governance also reinforced the prevalence of a communitarian lifestyle as the norm.

In this paper, we explored an alternative path to DSHR governance in Africa, which we termed decolonisation and self-regulation. Decolonisation is used not in terms of the historical colonial past, but rather to the act of rethinking and reshaping DSHR governance in Africa based on its unique societal, cultural, and ethical perspectives. We ask whether it is possible to approach data science health research from Africa’s unique perspective. Rather than adopting a framework predominantly shaped by Western paradigms, the paper proposes exploring whether Africa’s values, traditions, and communal priorities can inform and shape the governance of DSHR in a manner that is both relevant and respectful to local contexts. This approach invites a shift from merely adapting external models to actively crafting governance frameworks that emerge organically from African societies themselves. Self-regulation extends beyond the conventional idea of simply adhering to externally imposed rules. It suggests a more autonomous and self-determined approach, where African nations and communities take responsibility for the governance and ethical oversight of DSHR. This perspective argues that Africa’s distinct cultural, ethical, societal, and philosophical foundations provide an inherent capacity for self-governance that is both sustainable and culturally appropriate. By self-regulation, we do not mean an unchecked concentration of power in the hands of a few, but rather an ethic of restraint and accountability embedded within African communitarian traditions. In this sense, self-regulation complements, rather than replaces, formal legal frameworks. Health researchers must demonstrate judgment and responsibility in navigating the complexities of DSHR, particularly in decisions about the volume of data to collect, when to anonymise information, and whether publishing identifiable data serves a genuine public interest. Grounded in African communal values, self-regulation is reinforced by structures of collective accountability, such as peer oversight, community engagement, and relational ethics that mitigate risks of elite capture while ensuring that ethical responsibility remains at the center of research practice. Ultimately, it is indigenous moral frameworks such as *Ubuntu* and *Omoluabi* which emphasise human dignity, solidarity, and noble character that guide the model of self-regulation this paper proposes.

### Materials and methods

This paper adopts a case study methodology, employing a comparative analysis of decolonisation and self-regulation in health research across five African countries: Nigeria, Kenya, Ghana, Uganda, and South Africa, each representing their respective regions and actively engaged in global research collaborations. Countries from North Africa were excluded due to language limitations, as some key materials were not available in English. The study draws on doctrinal analysis of legal and policy frameworks, complemented by a review of peer-reviewed literature, government policy documents, ethics codes, and institutional guidelines. The study further draws on a diverse range of online resources and databases, including PubMed, Google Scholar, HeinOnline, and government websites. Additionally, it incorporates reviews of case law, social media pages, and external sources such as funders, networks, and scholarly articles.

### Current landscape of data science health research governance in Africa

The current landscape of DSHR governance in Africa is shaped largely by the already existing health research governance structures established over the past decades. Ethical frameworks, including Research Ethics Committees (RECs), have been instituted by many countries to review and oversee health research. RECs align their ethical review processes with international standards like the Declaration of Helsinki, the Council for International Organisations of Medical Sciences (CIOMS), the Belmont Report, the NIH Guidelines for the Conduct of Research Involving Human Subjects, as well as the World Health Organization (WHO) Operational Guidelines for Ethics Committees.

Research ethics regulations are fundamentally rooted in the Belmont Report, CIOMS Guidelines, and the Helsinki Declaration. These codes are anchored on the principle of autonomy, emphasising that individuals capable of making decisions must be allowed to do so, particularly in clinical care and research ^80^. Considering that the principle is usually a condition for the award of grants, this principle has been entrenched within the African research community. However, the emphasis on individual autonomy often clashes with communal decision-making norms in many African contexts, where choices affecting health and research participation are negotiated within families or communities. Interpretations of autonomy in these settings tend to be relational rather than individualistic. While some commentators from liberal individualist perspectives characterise communal decision-making as constraining individual freedoms, little empirical work has examined how autonomy is operationalised in contexts where communal decision-making predominates. Communal decision-making practices, while sometimes portrayed as limiting individual choice, are in fact deeply rooted in cultural norms that prioritise collective welfare and social harmony ^81– 84^. This cultural context contrasts with research ethics traditions that interpret autonomy primarily in individualistic terms. For example, practices that may be considered routine in many African communities, such as deferring to household heads or seeking approval from community leaders, would often be regarded as incompatible with prevailing ethical norms in more individualist-oriented settings. This underscores the importance of reconciling global research ethics principles with local cultural realities.

To address this gap, some African researchers began to emphasise community engagement. According to Tindana *et al.*, community engagement is the establishment of genuine partnerships characterised by mutual respect, inclusive participation, power sharing, equity, and shared benefits to seek a "win-win" outcome in collaborative initiatives ^85^. Community engagement is significant because it takes cognisance of community values, beliefs, and norms as they shape perceptions of study risks and benefits, influencing both independent decision-making and consent processes. Active community participation helps tailor consent procedures to local contexts, while engagement activities enhance communication, fostering respect, understanding of research implications, and acknowledgement of participants' contributions ^86, 87^.

Community engagement activities may also facilitate interpersonal communication, which is key to showing respect and making people understand the risks and benefits of the research, as well as appreciating participants' contributions ^88^. Through this approach, researchers argue that communities and their leaders must be involved and respected before any research is conducted, particularly in the demographic areas where the study will take place. Moreso, in an era of increasingly complex data ecosystems, where obtaining meaningful consent for online transactions is becoming impractical, the foundational emphasis on autonomy in research ethics guidelines warrants reconsideration. This is not a critique of autonomy but a call for reflection: if autonomy is the cornerstone of research ethics, it should not hinder the growth of DSHR. Researchers ought to insist on it even where the volume and complexities of the datasets present a challenge. However, the fact that one can argue that it can be dispensed with means that fundamentally, it is not an escape route ^7^. Africa’s experience suggests that the challenges autonomy presents in some communities should inform more inclusive frameworks.

Secondly, assuming that research ethics guidelines were fundamentally developed in Africa, would the concept of informed consent have evolved differently? While respecting research participants would remain a priority, the process might reflect communal values rather than the purely individualistic approach of the current model. Some studies suggests that acquiring voluntary consent in African cultures can be difficult, requiring more than just providing information and securing consent ^89– 93^. Religion, language hurdles, literacy levels, power disparities, and socio-cultural norms and beliefs can limit individuals' ability to freely agree to participate in research ^83, 84, 94^. These issues are exacerbated where research is conducted in communities with strong ties to religious and cultural norms or where teenagers are actively involved in the research ^95– 97^.

Finally, while scholars worldwide grapple with the challenges posed by DSHR, there is much to learn from Africa. Despite decades of adhering to external research ethics frameworks, African scholars have worked to preserve their cultural norms and practices through self-governance. This unique approach could serve as a model for integrating diverse perspectives into global research ethics frameworks.

### Case studies of decolonised research models in Africa

Within Africa, over 40 countries have adopted some form of research ethics codes, largely reflecting global standards such as the Belmont Report, CIOMS Guidelines, and the Helsinki Declaration. However, a select few countries and initiatives have begun incorporating localised and decolonised approaches into their health research frameworks. This section highlights and discusses examples of these efforts.

***The San People of Southern Africa.*** The San people of Southern Africa, also known as Bushmen, are one of the world’s oldest continuous cultures, with their history stretching back tens of thousands of years ^98^. They are indigenous to Southern Africa, with a cultural identity identified as hunter-gatherers, a shared ancestry that has now been confirmed by genetic research ^99^. Because they are the oldest genetic ancestors of modern humans, they have for years been the focus of health research ^100^. San leaders have often felt dissatisfied and distasteful about some of the research and conclusions reached, especially when the San people were the subjects of research ^101^. Consequently, in 2017, through strong leadership, the San leaders of Southern Africa developed the San Code of Research Ethics, the first of such in Africa. The key point contained in this Code is that researchers engaging with San communities must uphold the principles of fairness, respect, care, and honesty while also obtaining community approval through a simple process of community structures that were put in place ^102, 103^. The San Code emphasises respect not only for individuals but also for the community, culture, and history. It specifically highlights past instances where San leaders were disregarded and excluded from the research process. The Code calls for open and transparent communication between researchers and San leaders, ensuring meaningful engagement. More so, it stresses that research should align with local needs and contribute to improving the lives of the San people. The Code emphasise that this care must extend to the families of those involved, as ethical research should recognise and respect the San people's identity while adhering to the cultural and social principles outlined in the Code of Ethics. In South Africa, communal consent is frequently operationalised through Community Advisory Boards (CABs), which act as intermediaries between research teams and local communities. For instance, in a schizophrenia genomics study among Xhosa participants, researchers established a population-specific CAB that advised on recruitment, consent procedures, and stigma-related concerns ^104^. Similarly, biobanking projects have incorporated Ubuntu-informed models of engagement that emphasise solidarity and relational accountability ^105, 106^. These models include extended community consultations, participatory governance structures, and public feedback mechanisms, thereby reconciling the individual consent requirement with African communitarian expectations.

***Kenya.*** Kenya has implemented frameworks like the Kenya Medical Research Institute Guidelines (KEMRI), which prioritise local involvement and community consultation in health research.

Ethical approvals in Kenya often require researchers to demonstrate how they have engaged with local communities, reflecting an effort to align research with the values and needs of Kenyan society. For example, Section 9.1 of the KEMRI mandates investigators to ensure that community engagement, recruitment methods and advertisement for enrolment in a study are done in an ethically acceptable manner and that materials and methods used in recruitment are approved by KEMRI SERU ^107^. The guidelines equally made provisions for the community advisory boards, a group that presents the interests of the community concerning the planned research to the Principal Investigator (PI). The PI is expected to define their community engagement plan in the study protocol and recruitment procedures. This will document relevant stakeholders, how potential participants will be approached, incentives to be given, and materials and advertisements to be used. Most importantly, Section 11.1 of the guidelines provides that to mitigate the risk of the research, community leaders must be involved throughout the regular meetings of the research.

 Some researchers have relied on traditional assemblies and community forums to negotiate consent for studies involving vulnerable groups, such as children affected by HIV/AIDS. Vreeman *et al.* describe how community meetings, known locally as barazas, were used to gather collective views on participation, after which individual consent was sought from caregivers in private ^108, 109^. In a large-scale genomic studies of severe childhood diseases, researchers combined public meetings with consultations with village leaders, elders, and households to ensure both legitimacy and trust ^94^. This dual-layered approach respected cultural expectations for communal deliberation while preserving voluntariness at the individual level.

For the San people in particular, traditional structures, such as elders and community councils, are involved in the research process. For research conducted among indigenous communities or in rural areas, researchers are required to obtain the communal consent of leaders, elders, or councils before engaging individuals. This practice suggests that obtaining communal consent from leaders, elders, or councils is crucial for ethical research with indigenous communities where community decision-making is prioritised over individual autonomy. This also emphasises the importance of cultural sensitivity and collaboration within these communities ^110– 112^.

***Nigeria.*** Nigeria, the most populous country in Africa, is a nation of immense cultural and religious diversity. With over 250 native languages and multiple religious affiliations, health research in the country has often been fraught with controversy. However, since the introduction of the Health Research Ethics Code in 2007, greater coordination and orderliness have been brought to health research governance. As a predominantly paternalistic society, with over 50% of the population identifying as Muslim, traditional consent requirements have historically posed challenges ^113^. Individual consent for women was particularly difficult to implement, as male family members customarily provided consent on behalf of their wives, and fathers did so for their unmarried daughters ^114, 115^. This practice raises important considerations in the context of global health research ethics. The principle of autonomy asserts that individuals with decision-making capacity should be allowed to make choices concerning themselves. While religious and cultural factors may influence consent practices, a critical question arises: if a woman willingly permits her husband to make decisions on her behalf, has the woman exercised her autonomy? In a broader sense, if a community collectively agrees that a "king" should make decisions on their behalf based on their trust and belief in the ruler's character, can this be considered an exercise of their autonomy? These distinct African perspectives, although diverging from prevailing ethical norms in Western settings, warrant further engagement and recognition within global health research.

More broadly, the Nigerian Code of Health Research Ethics has institutionalised community engagement, mandating that researchers initiate discussions with communities before conducting studies. Ironically, this requirement places significant decision-making power in the hands of community leaders, who determine which research projects are acceptable for their members. To facilitate dialogue between community members, research participants, and researchers, the Code of Health Research Ethics provides for the establishment of Community Advisory Committees (CACs). These committees play a vital role in ensuring that community concerns are integrated into research activities. Some of their functions include relaying community concerns and issues to leaders, research teams, institutional officials, or the HREC, advising on key aspects of the informed consent process and assisting with participant recruitment and retention ^116^. By incorporating these mechanisms, the Research Ethics Code seeks to balance respect for cultural norms with ethical research practices, ensuring that health research in Nigeria remains locally relevant.

***Ghana.*** Ghana has actively implemented communal consent and community engagement strategies in health research, aligning research practices with traditional authority structures to ensure cultural appropriateness and community involvement. In northern Ghana, the Navrongo Health Research Centre (NHRC) has effectively integrated traditional community practices with modern research methodologies. By engaging local chiefs and elders through customary gatherings such as “durbars” the NHRC facilitates public discussions about proposed research projects. This approach not only respects traditional leadership but also fosters community trust and minimises potential ethical issues, thereby enhancing the ethical conduct of research. Similarly, the Kintampo Health Research Centre (KHRC) in central Ghana has prioritised community engagement as a core aspect of its research endeavours ^117^. The KHRC involves community members in defining research agendas and seeks their input throughout the research process. Oduro *et al.* report that research teams working in the Kassena-Nankana District sought initial permission from chiefs and household heads before approaching individual participants ^118^. This participatory approach ensures that studies address local health priorities and that findings are more readily accepted and implemented by the community ^117^. Additionally, the Global Health Research Unit on Global Surgery (GSU) in Ghana has collaborated with local communities to inform surgical research. By engaging hernia patients, community leaders, and other stakeholders in rural areas, the GSU has gathered valuable insights into the relevance, acceptability, and feasibility of clinical trials. This engagement has led to protocol adjustments that better reflect community needs and preferences ^119^. These initiatives underscore Ghana's commitment to integrating communal consent and community engagement into health research, ensuring that studies are culturally sensitive, ethically sound, and aligned with the health needs of its diverse populations.

***Uganda.*** Studies have shown that since gaining independence in 1962, Uganda has made concerted efforts to decolonise not only its political structures but also its health system governance ^120^. Scholars widely agree that colonial legacies in Uganda negatively impacted traditional forms of community participation in health system governance ^121, 122^. However, recent event shows that there are efforts to go back to the precolonial era. For example, the government is actively supporting traditional medicine practitioners, recognising their role in the health system, a practice that was previously suppressed under colonial rule. A significant turning point was the landmark case of *Salvatori Abuki and Richard Abuga v. Attorney General* ^123^, where the court declared unconstitutional the laws that criminalised traditional medicine practitioners under the guise of the Witchcraft Suppression Act. Additionally, initiatives such as *Bulungi Bwansi,* a self-help communal work system for public welfare, fostering collective action, traditional community engagement, and broader community participation, have been reintegrated into health system governance ^121^. Section 3 of the National Guidelines for Research Involving Humans as Research Participants makes provision for a CAB, which must include members such as religious leaders, community leaders, as well as individuals with an understanding of local laws, cultural laws and gender issues. The main function of the CAB is to assist investigators in understanding and incorporating community concerns into their research activities. Although the Guidelines specifically provide that community involvement should not override voluntary informed consent, the community advisory boards are empowered to advise on issues central to informed consent ^124^.

## Discussion

Taken together, these case studies demonstrate how values, traditions, and moral philosophies inherent in many African societies can shape research governance. However, their significance is best understood in relation to the broader practices outlined at the outset, particularly the models of PPI in the UK and CCI in Australia. In the UK, PPI has become an established requirement in research governance. Frameworks such as Involve Patients promote co-design and advisory panels to ensure that patients shape research priorities ^125, 126^. In Australia, the NHMRC requires demonstrable community engagement in ethics applications ^127^. These models emphasise accessibility, advisory mechanisms, and institutional participation. While valuable, they seem to primarily reflect individualistic traditions of consent, assuming that engagement occurs within literate, urban, and institutionally connected populations. In the UK, for example, *Involve* often recruits contributors via patient organisations, public outreach, and networks. These contributors are expected to participate in advisory groups, review draft research protocols, attend meetings, and comment on written materials. Researchers are encouraged to use plain-language summaries and accessible communication in engaging public contributors ^128^. Similarly, in Australia, the NHMRC consumer and community engagement is typically operationalised through public consultations, written submissions, and the involvement of consumer representatives (often via consumer or advocacy groups) on committees concerned with guideline development, peer review, and policy setting ^129^. It may be argued that these modes of participation presuppose the ability to read technical documents, navigate institutional processes, and engage in formal deliberation. In contrast, African models of engagement demonstrate how collective authority structures, and cultural negotiation can broaden the meaning of participation beyond the institutionalised and individualist assumptions embedded in the PPI and CCI.

More so, the manner and extent of PPI in some parts of the west differ significantly across contexts. For instance, in the UK, PPI is not only encouraged but mandated in publicly funded research, with structured frameworks guiding implementation. By contrast, recent evidence from health research more broadly shows that although public involvement is recognised in principle, its actual uptake remains low, inconsistently reported, and largely discretionary rather than compulsory ^130– 133^. Researchers are not obliged to embed public involvement in trial design, conduct, or dissemination, which often reduces engagement to a peripheral or symbolic exercise. For instance, Camelo *et al.* ^134^ indicated that stakeholders were not involved in the design and implementation of the study. Australia shows a clear gap between policy recognition and actual practice. National bodies strongly endorse consumer and community involvement, but in reality, the uptake has been limited. A systematic review, for instance, found that out of 325 randomized controlled trials published in the first half of 2021, only 17 (about 5%) reported involving the public in any way ^135^. This contrasts sharply with many African societies, where public or communal involvement is not an external mandate but an inherent cultural expectation. Engagement is woven into decision-making structures and communal ethics, such that excluding it renders research not only ethically deficient but socially void. In this sense, while some frameworks in the west attempt to institutionalise public involvement through regulation, African communitarian traditions embed it as a lived practice.

Over the past three decades in Africa, research ethics regulations have proliferated, with a strong emphasis on respecting research participants. Many African countries have established ethics codes, largely adapted from international frameworks such as the Belmont Report, CIOMS and the Declaration of Helsinki. Informed consent is a key requirement, and research ethics committees play a crucial role in ensuring its implementation. However, while informed consent is considered a fundamental prerequisite for ethical research, it remains unclear how this consent is obtained in practice, particularly in rural communities where community leaders hold decisive authority.

The San Code of Research Ethics in Southern Africa provides one of the clearest examples of a locally grounded approach. It emphasises community approval and respect for culture, with meaningful engagement requiring open and transparent communication between researchers and San leaders. The Code arose from a history of exclusion and exploitation, where researchers often communicated in technical scientific language that the San could not easily understand. Consequently, the San expect researchers to communicate in accessible language and show respect for cultural dignity. This emphasis on linguistic and cultural accessibility has broader resonance. In Nigeria, for instance, the failure to meet parts of the Millennium Development Goals was linked to inadequate communication in indigenous languages: essential informational booklets were not translated, leaving rural communities unable to understand health interventions intended for them ^136^. This further emphasises that, regardless of the good intentions behind global health practices, they must align with the cultural norms and values of the communities they aim to serve.This strong linguistic and cultural framing may contrast with practices in the UK PPI and Australian NHMRC guidelines which also stress accessibility and inclusion, but largely within literate, urban populations, thus, assuming that individuals can engage directly with institutional processes. Some of the African experiences demonstrate that meaningful participation requires adapting research into local cultural and linguistic forms.

Nigeria's advisory committees, as well as, and Kenya’s CABs further illustrate the integration of local norms into research governance. These bodies aim to align health research with community values by ensuring that experts who are socially connected to the population serve as intermediaries. At the KEMRI, for instance, community leaders are expected to be involved in regular meetings as a mechanism to mitigate risks and build trust. In northern Ghana, engagement with chiefs and elders through customary gatherings institutionalises this process, reinforcing legitimacy by embedding research within traditional authority structures. This aims to respect traditional leadership and foster community trust. Similar structures echo in the UK PPI where panels are convened to safeguard community interests. Again, a key difference lies in how tensions are resolved. While the UK and Australian models may rely on formalised institutional review procedures through Research Ethics Committees in the UK and Human Research Ethics Committees in Australia, African approaches often resolve tensions through dialogue with cultural authorities and negotiated consensus, reflecting communitarian traditions ^42, 137^. In the case where researchers first sought approval from local chiefs before contacting participants, potential tensions were addressed by ensuring that individual parents were separately informed and retained the right to decline their child’s participation without any negative consequences ^93, 118^.

While there are concerns that this gatekeeping risks undermining autonomy, more recent urban studies highlight that literacy barriers and household power dynamics continue to shape how consent is understood and practiced, particularly in low-income communities where community leaders play gatekeeping roles ^138^. Nevertheless, empirical studies across Africa demonstrate that while traditional leaders often serve as initial gatekeepers, African research ethics practice incorporates mechanisms to prevent undue concentration of authority. In Uganda, CABs in HIV vaccine trials balanced the input of local leaders with representation of youth and people living with HIV ^139^. In South Africa, H3Africa genomic projects developed layered engagement strategies involving chiefs, churches, and youth groups ^86^. Similarly, in rural northern Nigeria, researchers merged family head consent with individual assent to navigate both communitarian and individual expectations. Genomics research in Ghana involved local chiefs at first, however, researchers supplemented chiefly approval with open durbar meetings to ensure younger voices were heard ^93^. In Kenya, community engagement committees explicitly include elders, youth, and professionals to prevent unilateral decision-making ^140^. These examples illustrate how African approaches to consent not only rely on communal values but also evolve practical checks against over-centralisation of authority.

Unlike in many African contexts where scholars call for the decolonisation of health research governance largely from within academia, Uganda provides a notable example of governmental leadership. The Ugandan state has taken concrete steps to embed decolonised principles into law and practice, through case law, legislative reform, and community-based initiatives such as the *Bulungi Bwansi*. This approach moves beyond advisory functions to embed ethical governance into the broader legal-political system. South Africa’s Good Clinical Practice Guidelines provide another example of blending global standards with local realities. Overseen by the National Health Research Ethics Council (NHREC), the framework incorporates community engagement, cultural sensitivity, and respect for local norms ^141^. Importantly, research involving indigenous knowledge and traditional medicine is subject to regulation designed to protect cultural heritage, reflecting a move toward decolonised practices that preserve local ownership. Similarly, in Botswana, community participation is institutionalised through the involvement of tribal authorities in research approval processes. In essence, tribal leaders must provide consent on behalf of their communities before research can proceed, reflecting a communitarian rather than individualist model of consent ^142^. The Botswana Health Research and Development Committee requires that research demonstrate relevance to community needs, while traditional leadership structures play a formal role in ensuring alignment with local values.

Placed alongside some of the engagement frameworks in the west, the African case studies reveal both parallels and divergences. While the UK’s PPI and Australia’s CCI guidelines promote patient and public involvement, it can be argued that participation privileges those with higher literacy and institutional access. By contrast, African practices highlight that community consent and traditional authority can carry equal, or even greater, legitimacy than individual consent, particularly in rural or indigenous settings where communal structures are socially authoritative ^39, 43, 143^. Admittedly, both models risk marginalising certain groups, though in different ways: in the UK and Australia, exclusion often stems from formal literacy and professionalisation, while in African contexts it is shaped by reliance on cultural authority. While not without challenges, the latter reflects a normative commitment to communitarian legitimacy, rather than a pragmatic response to barriers such as literacy.

Overall, while these case studies, inclusive of the examples from the UK and Australia demonstrate diverse approaches to governance, they do not by themselves resolve the normative question of what ought to be done. International and regional instruments such as the Universal Declaration of Human Rights, 1948 and the African Charter on Human and Peoples’ Rights, 1981 articulate commitments to dignity, equality, and non-discrimination that can be used to assess whether particular practices protect or undermine these standards. Similar principles are echoed in the Universal Declaration on Bioethics and Human Rights, 2005, which directly links human rights to ethical guidance in biomedical and health research. Applying a human-rights lens requires critically interrogating customary practices where they risk excluding or harming vulnerable groups. For example, while the North-western Nigeria deference to the male head of household or to community approval can foster legitimacy and trust, it may also marginalise women, young people, or other groups, raising potential conflicts with rights-based obligations to equality and participation. Ethical deliberation must therefore recognise what is done in practice while asking whether those practices conform with minimum rights-based protections and, where necessary, identifying reforms that preserve cultural legitimacy without violating fundamental rights ^144^.

There is often a natural tension between community self-regulation and individual human rights. On one hand, self-regulation works because it draws strength from local values, shared norms, and collective decision-making. But this can sometimes mean that the rights of individuals are not fully protected. On the other hand, if governance is based only on individual rights, it can weaken the authority of the community and reduce trust, especially in places where collective responsibility is highly valued. When the gatekeeping role of the community is overlooked, research itself may also suffer, since projects that ignore communal authority risk rejection, low participation, or lack of long-term support. A balanced approach would be protecting the dignity and equality of individuals, while also respecting the role of community structures. Human rights should not be seen as conflicting with self-regulation; rather, they provide the guiding framework that ensures self-regulation is fair, legitimate, and sustainable.

### What does this mean for DSHR?

Data science health research raises ethical challenges that stretch the traditional model of informed consent. In many African contexts, these challenges may manifest differently, as decision-making is frequently embedded in collective structures and mediated through trusted community leaders. However, the absence of strict individual consent does not necessarily mean that legitimacy is lost. In societies where people tend to think in terms of “we” rather than “I,” the authority of the group and the practice of reaching consensus can provide a form of social cohesion that individual consent alone cannot achieve. For DSHR, this means that consent should not be treated as a uniform or universal requirement. Individual rights are crucial, but they need to be complemented by community oversight and accountability structures that resonate with local traditions. These communitarian practices are a principled way of recognizing that data belongs to people collectively, not only individually, and that decisions about its use can rightly be made together. This perspective is particularly important in data science, where risks are often collective. Entire groups can be stigmatised, re-identified, or disadvantaged by the way data are combined and analysed. Involving communities in governing such risks is ethically sound, but it must be accompanied by safeguards, clear rules, transparent processes, and technical protections, that defend both individuals and groups

## Conclusion

The evolving landscape of DSHR calls for a governance model that is both ethically sound and culturally relevant. Informed consent has long been regarded as a cornerstone of global health research ethics because it embodies the principle of respect for persons. Yet, in practice, its dominant formulation places disproportionate weight on individual autonomy, which may not always align with the collective or relational values emphasised in many African societies. The challenges posed by DSHR, particularly regarding the feasibility of obtaining individual consent in large-scale data use, further expose the limitations of this governance model. However, these challenges should not be viewed as obstacles but rather as an opportunity for the global research community to fashion out governance structures that align with their indigenous ethical perspective. The frameworks emerging in countries such as Nigeria, Ghana, Kenya, and Uganda, in addition to the San Code of Research Ethics in Southern Africa, demonstrate a shift toward decolonised and self-regulated approaches that prioritise community engagement, respect for elders, and the role of local gatekeepers.

By embracing self-regulation and community-centred ethical standards, Africa has the potential to lead a transformative shift in global research ethics, which acknowledges multiple pathways to ethical legitimacy and affirms the value of diverse cultural and philosophical traditions, rather than relying on a single, uniform model. A decolonial and plural approach to informed consent and research ethics in global health can enhance social justice and accountability for all stakeholders ^145^.

## Limitations of this study

Decolonisation is not merely a theoretical concept, but a practical and necessary process that requires deliberate action. Those committed to decolonisation must take proactive steps to address the challenges that arise when dependence on Western structures is rejected. However, African governments have repeatedly demonstrated an inability or unwillingness to engage in meaningful discussions and initiatives on decolonisation. Despite the continent's vast resources, many political leaders continue to rely on foreign aid and grants from Western nations rather than pursuing innovative, homegrown solutions to economic challenges. Instead of fostering self-sufficiency, this dependency perpetuates poverty and hinders sustainable development. While private institutions and researchers are making commendable efforts to advance the decolonisation agenda, policy discussions in this area ultimately require political will. Since legal frameworks largely dictate national policies and institutional practices, any substantial progress must be driven by legislative and governmental action. Unless African leaders take decisive steps to restructure governance, prioritise self-reliance, and invest in homegrown research and development, the discourse on decolonising health research in Africa will remain purely theoretical, lacking the practical implementation needed for real change.

Secondly, this study acknowledges that limiting the analysis to five countries out of Africa’s 54 may not fully capture the continent's diverse health research governance landscapes.

## Further research

While this paper outlines the need for decolonised approaches to health research governance, particularly as it concerns DSHR, further research is required to develop concrete methodologies and procedures for implementing these proposals effectively. Further research could explore more case studies within diverse African sociopolitical contexts to assess best practices for integrating Africa’s unique communitarian ethos into existing regulatory structures in a way that is globally accepted and ethical.

## Ethical approval

This research study did not involve human participants, identifiable personal data, or biological samples and therefore did not require ethical approval.

## Consent

N/A

## Data Availability

The data for this article consists of bibliographic references, which are included in the References section. N/A

## Supplementary materials

N/A
