# Supplementary material for: Decolonisation and Self-Regulation as Alternative Paths to Data Science Health Research Governance in Africa
Source: Wellcome Open Res. 2025 Dec 11;10:380. Originally published 2025 Jul 30. [Version 2] doi: 10.12688/wellcomeopenres.24070.2 (PMC12853021; doi:10.12688/wellcomeopenres.24070.2)
Supplement: Peer review history

# Reviewer report: Reviewer response for version 2

**DOI:** 10.21956/wellcomeopenres.27741.r142306

**Published:** 2026-01-28

**Authors:** Pier-Luc Turcotte

**Affiliations:** 1 University of Ottawa, Ottawa, Ontario, Canada

I would like to thank the authors for writing this important piece that seeks to provide an alternative to western dominant models of ethical frameworks. I find the case studies to be informative and detailed and they provide a guidance for undertaking these alternative approaches rooted in decoloniality.  Apart from the clarity and substantiality of the argument, I particularly appreciate how this paper challenges the dominant individualistic views of informed consent, which are inherently detaching individuals from the context in which decisions are made. This view reinforces colonial and imperialistic dynamics when applying western approaches to bioethics.  This study has relevance for any research conducted within Indigenous contexts beyond Africa. In Canada, First Nations community often define confidentiality as a colonial principle which distances research practices from collective understanding and ownership of knowledge. As the authors highlight, Africa bears the consequences of colonial and imperialistic practices, leading it to bear a quarter of the global disease burden. This in-itself should justify applying ethical principles rooted in indigenous ways of knowing and doing.  My only minor request would be to revise the sentence beginning with: "According to Frantz Fanon, the proponent of the Fanonian approach". Consider removing "the proponent of the Fanonian approach". Fanon is not the proponent of his own approach which outlived him. This portion is implied and not necessary. I hope this paper is indexed so I can refer to it in my own research. Thanks to the authors for producing it.

Is the work clearly and accurately presented and does it cite the current literature?

Yes

If applicable, is the statistical analysis and its interpretation appropriate?

Yes

Are all the source data underlying the results available to ensure full reproducibility?

Yes

Is the study design appropriate and is the work technically sound?

Yes

Are the conclusions drawn adequately supported by the results?

Yes

Are sufficient details of methods and analysis provided to allow replication by others?

Yes

Reviewer Expertise:

My research is conducted with First Nations communities in Canada. I apply Fanon's work and other anti-colonial approaches in health and rehabilitation.

I confirm that I have read this submission and believe that I have an appropriate level of expertise to confirm that it is of an acceptable scientific standard.

# Reviewer report: Reviewer response for version 2

**DOI:** 10.21956/wellcomeopenres.27741.r143899

**Published:** 2026-01-24

**Authors:** Yann Joly, Jessica Huang

**Affiliations:** 1 McGill University, Montréal, Canada; 2 Victor Phillip Dahdaleh Institute of Genomics Medicine, McGill University, Montreal, Canada

Thank you for the opportunity to review this manuscript. This is a conceptually ambitious and timely piece that engages an underexplored question: how data science health research (DSHR) governance might be rethought through African communitarian ethics and decolonial perspectives. The writing is generally clear and easy to follow, the authors provide a helpful overview of why DSHR strains traditional informed consent models, and the amendments addressing previous reviewers’ comments offer substantial improvements. I recognize that my perspective is shaped by training in research ethics outside the Global South, which may introduce limitations or bias in how I interpret communitarian governance models and decolonisation arguments.

 Major comments

1. **Scope and specificity of DSHR governance claims** – One central concern is that the authors at times conflate challenges associated with research conducted without individual consent in general with challenges specific to DSHR. The paper does not make clear whether the proposed communitarian governance approach resolves DSHR-specific ethical issues (e.g. secondary data use, automated decision-making, cross-border data flows, continuous learning AI systems, group-level harms, re-identification risks), or whether it primarily addresses traditional informed consent limitations. It should be considered, for example, how communitarian frameworks address algorithmic group harm, what governance mechanisms suit continuous data reuse, or how collective consent function when data subjects are unaware their data exists in research databases. If this DSHR-specific issues are indeed the focus of the piece, it would be helpful to clarify how African governance frameworks meaningfully respond to distinct DSHR challenges beyond consent, otherwise the title should be revised to reflect broader health research governance with DSHR as illustrative.
2. **Engagement with rights-based critiques** – Earlier reviews of the piece raised concerns about concentrated authority and individual rights violations, and the revision acknowledges these issues but doesn’t resolve them. The authors state that they must be balanced (p.10), but don’t explain how to balance them when they conflict, such as providing a framework for resolving internal dissent or addressing discriminatory practices (e.g., male heads of household consenting on behalf of women). Adding concrete governance mechanisms, such as mandatory consultation with potentially marginalized groups, individual opt-outs, or data transparency would substantially strengthen this section.
3. **Distinctiveness of the proposed governance alternative** – The paper positions African communitarian governance as complementary to, rather than a replacement for, existing models such as UK PPI, Australian CCI, Indigenous CARE principles, and First Nations data sovereignty frameworks. However, it remains unclear what new normative principle, governance mechanism, or practical decision rule this proposed approach introduces that would substantively change DSHR governance beyond existing models. The inclusion of Ubuntu's ontological foundation and Omoluabi's character-based ethics adds valuable conceptual depth but adding how these philosophies are practically applied would strengthen the argument.

Minor comments

1. Methodology (p.6): The authors should provide a more detailed rationale for selecting the five case-study countries. The exclusion of North Africa due to language limitations can be explained further or can be acknowledged as a potential bias in limitations.
2. Decolonisation (p.5-6): The term “decolonisation” seems to serve a dual purpose: historical independence and reshaping governance based on African perspectives. The authors could consider using distinct terms (“political decolonisation” vs “epistemic decolonisation”) and clarify whether this means African data sovereignty, rejecting Western frameworks of data consent, developing hybrid models, or something else.
3. Self-regulation (p.6, Discussion): The discussion of self-regulation risks conflating researchers' moral agency with community governance structures. The authors should clarify whether "self-regulation" refers to researcher discretion, community self-governance, or African states regulating without external frameworks.
4. The authors should clarify if there is a mechanism to resolve situations in which external actors (Global North researchers, industry) may exploit community consent within communitarian frameworks to bypass individual protections or justify extractive data practices.

Is the work clearly and accurately presented and does it cite the current literature?

Yes

If applicable, is the statistical analysis and its interpretation appropriate?

Not applicable

Are all the source data underlying the results available to ensure full reproducibility?

No source data required

Is the study design appropriate and is the work technically sound?

Yes

Are the conclusions drawn adequately supported by the results?

Partly

Are sufficient details of methods and analysis provided to allow replication by others?

Partly

Reviewer Expertise:

ELSI of genetics, data science ethics, research ethics, data sharing

We confirm that we have read this submission and believe that we have an appropriate level of expertise to confirm that it is of an acceptable scientific standard, however we have significant reservations, as outlined above.

# Reviewer report: Reviewer response for version 1

**DOI:** 10.21956/wellcomeopenres.26556.r128832

**Published:** 2025-09-05

**Authors:** Melissa McCradden

**Affiliations:** 1 The University of Adelaide, Adelaide, South Australia, Australia

This is a thought-provoking and challenging piece, which I think is a valuable contribution to the literature. The paper describes use case examples of the application of decolonization applied to the issue of consent in data science health research within the African continent. This reviewer offers some major and minor recommendations for consideration of improving the article's rigour.

 MAJOR

 As indicated below, it is unclear if the fit for this paper is in the 'Original Research' category as it is more accurately describes as a case study (as noted in the Materials and Methods section). This would validate the authors' approach which is non-systematic in nature and does not require comprehensive reporting of methodology.

 It would be valuable to see the paper engage further with the extensive literature around patient and public engagement in 'Global North' or globally dominant countries (E.g., UK and Australia in particular). The practices described in the use cases are very closely matched to the requirements around research in the Australian NHMRC, for example. Certainly, this comment does mean to say that the approaches in these countries are the standard or are effective; rather, it would be of great value to the literature for readers to understand the uniquely African contributions that expand how we should understand engagement frameworks for research. Particularly, it would be interesting to see differences in how not only the concepts are applied, but how the tensions are resolved (further comments below).

 “For instance, a decision that might seem routine in Northwestern Nigeria, such as deferring to the male head of the family, a husband or getting community approval, might be seen as outrageous in the more advanced economies such as the United States or the United Kingdom.” I wonder if the authors might consider the potential relevance of human rights in a context like this, which many have used to ground certain required commitments within the cultural context? In bioethics, for example, it is long recognized that simply because a situation is the case, does not necessarily mean it ought to be the case. Within both Western and non-Western contexts considering what IS done against what SHOULD be done is a core feature of ethical deliberation, and grounding in human rights provides a common foundation on which to build. It would be valuable to understand how these sorts of perceived universal concepts apply (or perhaps do not apply?) within this context?

 Along similar lines, it would be helpful to articulate the authors’ reflections on the argument that rather than being decolonial, communalist approaches to consent are at risk of recapitulating colonialism by consolidating power in the hands of a few. These few then decide what the best thing is for a larger group of individuals. This concern may be particularly salient in light of the fact that community and patient engagement research in the context of data science has been criticized for being tokenistic and symbolic (e.g., Madden & Speed, 2017). How might we address the tensions of self-regulation and human rights? The authors touch on some of the tensions between individual rights and communal benefit in the paper, and it would strengthen to paper to see more engagement on this topic.

 As the topic is about data science governance, it might be worth expanding whether there is something unique about data science and its considerations (or is there not?) and the implications of such under these frameworks.

 MINOR

 Section 1 - in patient engagement, an often utilized framework is that of the ladder, wherein the level of engagement and the reciprocity required are considered. Eg, at the lowest level, ‘inform’ is utilized whereas the highest level is empower (patient has control over the whole research). Does this apply in the authors' own context, or is engagement an all-or-nothing conceptualization?

 In the abstract, the Methodology section states 'mixed-methods' as an approach; however, no formal methodological framework relevant to mixed methods is applied. The notion of a 'case study' is more accurate and applicable, and does not devalue the importance of this paper. If this framework is applied, it is thus not a problem that the paper is lacking in methodological details.

 A key piece I think the authors might wish to consider is how to reconcile some of discrepancies between young and older generations in terms of the norms they wish to abide by. For example, a potential vulnerability of requiring the approval of a local elder or king before the community is approached about research rests a considerable amount of power in a single person. And people - of all races - are subject to their own beliefs and values, which means that potentially beneficial research may not reach certain communities, depriving them of access-related opportunities. How might African approaches to consent suggest a researcher manage such a tension?

Is the work clearly and accurately presented and does it cite the current literature?

Partly

If applicable, is the statistical analysis and its interpretation appropriate?

Not applicable

Are all the source data underlying the results available to ensure full reproducibility?

No source data required

Is the study design appropriate and is the work technically sound?

Partly

Are the conclusions drawn adequately supported by the results?

Partly

Are sufficient details of methods and analysis provided to allow replication by others?

Partly

Reviewer Expertise:

bioethics, research ethics, data science

I confirm that I have read this submission and believe that I have an appropriate level of expertise to state that I do not consider it to be of an acceptable scientific standard, for reasons outlined above.

# Reviewer report: Reviewer response for version 1

**DOI:** 10.21956/wellcomeopenres.26556.r129872

**Published:** 2025-09-04

**Authors:** Abdoul Jalil Djiberou Mahamadou

**Affiliations:** 1 Stanford University, Stanford, CA, USA

I appreciate the opportunity given by the authors and editor to review the manuscript. The authors advocate for localized and culturally sensitive data science in health research governance in Africa, with a particular emphasis on informed consent. The paper is timely, as artificial intelligence (AI) is increasingly being adopted in Africa, particularly in healthcare and medicine. African countries are also aiming to establish regional and national governance measures, as highlighted in the African Union's Continental AI Strategy. I appreciate the representativeness of the case studies as related to different African regions.

 I have a few comments about the manuscript:

1. The authors state “[DSHR] presents new ethical challenges to the traditional model of human subject research, particularly by enabling data processing **without the consent of data subjects**.” Research can be justified without consent under a waiver of consent. For instance, in the US, section 45 CFR 46.116(c) specifies conditions under which consent can be waived. Thus, the authors should make a distinction between researchers who have a waiver of informed consent and those who don’t.
2. The authors should make distinctions between traditional informed consent (e.g., for data collection and use) and consent as disclosure of the use of AI in the clinical setting.
3. The authors situate the manuscript title and introduction within the umbrella of data science and health research. However, the case studies and discussion appear to be independent of data science. For instance, the NHRC has existed for over three decades, and I assume their practices around informed consent. Broadly speaking, some aspects of African human research governance may have existed even before the advent of modern data science. I suggest the authors discuss how modern data science challenges the elements of [African] informed consent/governance (i.e., community engagement, cultural sensitivity, and respect for local norms).
4. The authors should make distinctions between the self-regulation as defined in the manuscript and the self-regulation terminology used in the literature. For instance, studies have shown that healthcare AI researchers are self-regulated in the sense that they may or may not have a moral engagement with the AI systems they develop (see PMC9782696 and PMC10690528). Although the mentioned study is not specifically in the context of informed consent and Africa, its findings can be extrapolated to these contexts. In line with the authors' self-regulation definition, “Self-regulation implies that individuals can exercise a degree of control over their affairs, rather than relying solely on established legal rules,” what if African researchers decide to conduct research without participants’ consent?
  I do not quite agree with this on page 6: “This includes making informed decisions about the volume of data to use, determining when to anonymise data, and assessing whether publishing identifiable information is justified, particularly when it does not serve the public interest”. This implies that a researcher, particularly one with a power imbalance, can collect unnecessary data and inadvertently expose participants’ identities.
5. The authors should acknowledge that not all Western governance initiatives are individualistic. For instance, indigenous data governance in the US and Canada (e.g., https://www.gida-global.org/care) has some overlapping principles (e.g., collective well-being) with African cultures and traditions.
6. There won’t be a one-size-fits-all model of informed consent in the context of DSHR. For instance, informed consent in genomic research processes may differ from that in other domains. For more information, I recommend consulting the work of Dr. Jantina de Vries (https://scholar.google.com/citations?user=aT-TJQgAAAAJ&hl=en&oi=ao) or the H3Africa consortium (https://h3africa.org/), which has published extensively on topics such as informed consent, community engagement, and other ethical issues in Africa. Thus, I suggest that future iterations of the work on developing “concrete methodologies and procedures for implementing these proposals effectively” focus on specific data types or AI applications.
7. There is a significant lack of literature on decolonial data and AI governance in Africa, for instance, using the Ubuntu Philosophy and specifically related research ethics and informed consent, which I encourage the authors to acknowledge and discuss how their contributions differ from existing work. To name a few (see the added citations 1-5).

Is the work clearly and accurately presented and does it cite the current literature?

Partly

If applicable, is the statistical analysis and its interpretation appropriate?

Not applicable

Are all the source data underlying the results available to ensure full reproducibility?

No source data required

Is the study design appropriate and is the work technically sound?

Yes

Are the conclusions drawn adequately supported by the results?

Yes

Are sufficient details of methods and analysis provided to allow replication by others?

Yes

Reviewer Expertise:

Bioethics; AI Ethics; Computer Science; Cognitive Aging; Uncertainty Modeling

I confirm that I have read this submission and believe that I have an appropriate level of expertise to confirm that it is of an acceptable scientific standard, however I have significant reservations, as outlined above.

## References

[1] : The Ubuntu Way: Ensuring Ethical AI Integration in Health Research. *Wellcome Open Research* .2024;9: 10.12688/wellcomeopenres.23021.1 10.12688/wellcomeopenres.23021.1 PMC1159980239606617

[2] : An African Relational Approach to Healthcare and Big Data Challenges. *Science and Engineering Ethics* .2021;27(3) : 10.1007/s11948-021-00313-w 10.1007/s11948-021-00313-w 34047844 PMC8160550

[3] : Introducing Responsible AI in Africa. 10.1007/978-3-031-08215-3_1 1-11 10.1007/978-3-031-08215-3_1

[4] : Data revolution, health status transformation and the role of artificial intelligence for health and pandemic preparedness in the African context. *BMC Proceedings* .2021;15(S15) : 10.1186/s12919-021-00228-1 10.1186/s12919-021-00228-1 34809624 PMC8607787

[5] : African Data Ethics: A Discursive Framework for Black Decolonial AI. FAccT '25: Proceedings of the 2025 ACM Conference on Fairness, Accountability, and Transparency.2025;
